# Painless Acute Type A Aortic Dissection Masquerading as an Acute Neurological Event: A Case Report

**DOI:** 10.7759/cureus.104626

**Published:** 2026-03-03

**Authors:** Pacelli C Osigwe, Ogheneruno E Ozomaro, Ifunanya S Osigwe, Ebubechi Osigwe, Linda Uzoma-Azoma

**Affiliations:** 1 Stroke Unit, University Hospital of Wales, Cardiff, GBR; 2 Department of Medicine, Bronglais General Hospital, Aberystwyth, GBR; 3 Department of Medicine, University Hospital of Wales, Cardiff, GBR; 4 Department of General Practice, Improving Health Medical, Wolverhampton, GBR; 5 Department of Medicine, Tameside General Hospital, Ashton-under-Lyne, GBR

**Keywords:** acute type a aortic dissection, altered consciousness, altered mental status, aortic dissection, aortic emergency, case report, cerebral malperfusion, diagnostic delay, neurological event, painless aortic dissection

## Abstract

Acute aortic dissection (AAD) is a life‑threatening cardiovascular emergency that classically presents with sudden, severe chest, back, and/or abdominal pain. Painless presentations are uncommon and frequently lead to diagnostic delay. A 68‑year‑old man presented with an hour‑long episode of altered consciousness without chest pain or other typical symptoms. Neurological examination revealed no focal deficits, and cardiovascular assessment showed mild bradycardia and low‑normal blood pressure, attributed to his antihypertensive therapy, which included a rate‑limiting agent. His computed tomography head scan was unremarkable, and abnormalities on his electrocardiogram and chest radiograph were attributed to longstanding hypertension. He was admitted for observation with a presumed acute neurological event, possibly a non-convulsive seizure with a post-ictal state. Several hours later, oxygen desaturation prompted a computed tomography pulmonary angiography, which unexpectedly revealed an extensive type A aortic dissection extending from the aortic root to the level of the renal arteries. He underwent emergency aortic root and hemiarch replacement, metallic aortic valve replacement, and reimplantation of the coronary buttons (Bentall procedure). His postoperative course was complicated by multiple bilateral embolic brain infarcts and seizures, requiring prolonged stroke rehabilitation. This case highlights the diagnostic challenge of painless AAD, in which end‑organ complications such as cerebral malperfusion may dominate the clinical picture and delay recognition. To avoid potentially catastrophic delays in diagnosis, clinicians should maintain a high index of suspicion for AAD in patients presenting with acute or sudden‑onset neurological symptoms or signs, even in the absence of pain, particularly when initial assessment does not confidently establish any of the more common neurological or cardiac diagnoses.

## Introduction

Aortic dissection is characterised by a pathological tear within the aortic wall, typically in the tunica media, which allows blood to enter a false lumen separated from the true lumen by an intimal flap. A dissection is considered acute when symptom onset occurs within 14 days [1]. Acute aortic dissection (AAD) is a relatively uncommon but life‑threatening cardiovascular emergency, with an estimated incidence of 2.6-3.5 cases per 100,000 person‑years [1]. The Stanford classification divides AAD into type A, involving the ascending aorta, and type B, confined to the descending aorta. Classically, AAD presents with sudden, severe chest, back, and/or abdominal pain. However, a small proportion of patients, approximately 6.4%, remain entirely pain‑free, and in such cases, the presenting features of complications may dominate the clinical picture [2].

The mortality rate of AAD is high, increasing by an estimated 1-2% per hour from symptom onset in untreated cases [3]. Studies from Western Europe report 30‑day mortality rates ranging from 23% to 55.8% [4-6], and a nationwide retrospective study in Iceland found that nearly one‑fifth of patients died before reaching medical care [4]. Prompt diagnosis and treatment are therefore critical. Despite this, AAD is frequently misdiagnosed: up to 38% of cases are missed at initial evaluation [7], and a significant proportion are only identified at autopsy [8]. Painless presentations pose particular diagnostic difficulty and are associated with a higher risk of missed or delayed diagnosis, leading to poorer outcomes [2,8,9].

We report the case of a 68‑year‑old man who presented with altered consciousness in the absence of pain and was subsequently found to have an acute type A aortic dissection (TAAD) diagnosed incidentally after a delay. Reports of similar painless presentations of AAD are reviewed in the discussion.

## Case presentation

A 68‑year‑old man was brought to the emergency department (ED) shortly after midnight with altered consciousness. Less than an hour earlier, he had suddenly become vacant and unresponsive while sitting in a chair at home. His daughter, a healthcare worker, initiated chest compressions when she was unable to palpate a carotid pulse. These were discontinued within a minute when he abruptly regained responsiveness, although he remained disoriented.

In the ED, he was assessed by the medical emergency team. He was alert but disoriented, with a Glasgow Coma Scale (GCS) score of 14/15 (best eye response: 4; best verbal response: 4; best motor response: 6). His vital signs included a respiratory rate of 18 cycles per minute, oxygen saturation of 99% on room air, heart rate (HR) of 50 beats per minute, blood pressure (BP) of 102/41 mmHg, and temperature of 36.4 °C. Aside from the mild bradycardia, low-normal BP, and wide pulse pressure (61 mmHg), his cardiovascular, respiratory, and abdominal examinations were unremarkable. He was able to follow commands, and a detailed neurological examination revealed no focal deficits or signs of meningeal irritation. He denied pain, and his assessment did not include checks for peripheral pulse deficits or systolic BP differences between his limbs.

Approximately 20-30 minutes after arrival, he became fully alert and oriented, although he had no clear recollection of the events. The total duration of altered consciousness was estimated at around one hour. He continued to deny chest pain, dyspnoea, palpitations, dizziness, blackouts, focal neurological symptoms, or headache. There were no witnessed stiffening, jerky limb movements, abnormal eye movements, tongue biting, or incontinence. He had been well before the unresponsive episode, with no history of trauma, fever, chills, rigors, sweats, neck stiffness, photophobia, alcohol use, substance use, or drug overdose. His medical history included essential hypertension, ureteric calculi, Gilbert syndrome, and basal cell carcinoma. His regular medications were prolonged-release diltiazem (300 mg once daily), irbesartan (300 mg once daily), and hydrochlorothiazide (12.5 mg once daily), and he had no known drug allergies.

**Figure 1:**
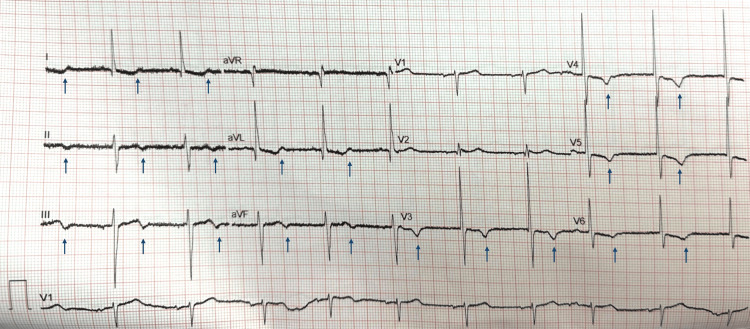
Electrocardiogram at hospital admission. An electrocardiogram recorded at admission showing sinus rhythm, large QRS voltages, and repolarisation abnormalities (arrows) in the anterolateral and inferior leads. These findings may indicate left ventricular hypertrophy and a left ventricular ‘strain’ pattern caused by longstanding systemic hypertension. However, in the absence of previous electrocardiograms for comparison, the repolarisation abnormalities (arrows) may also represent myocardial ischaemia.

**Figure 2:**
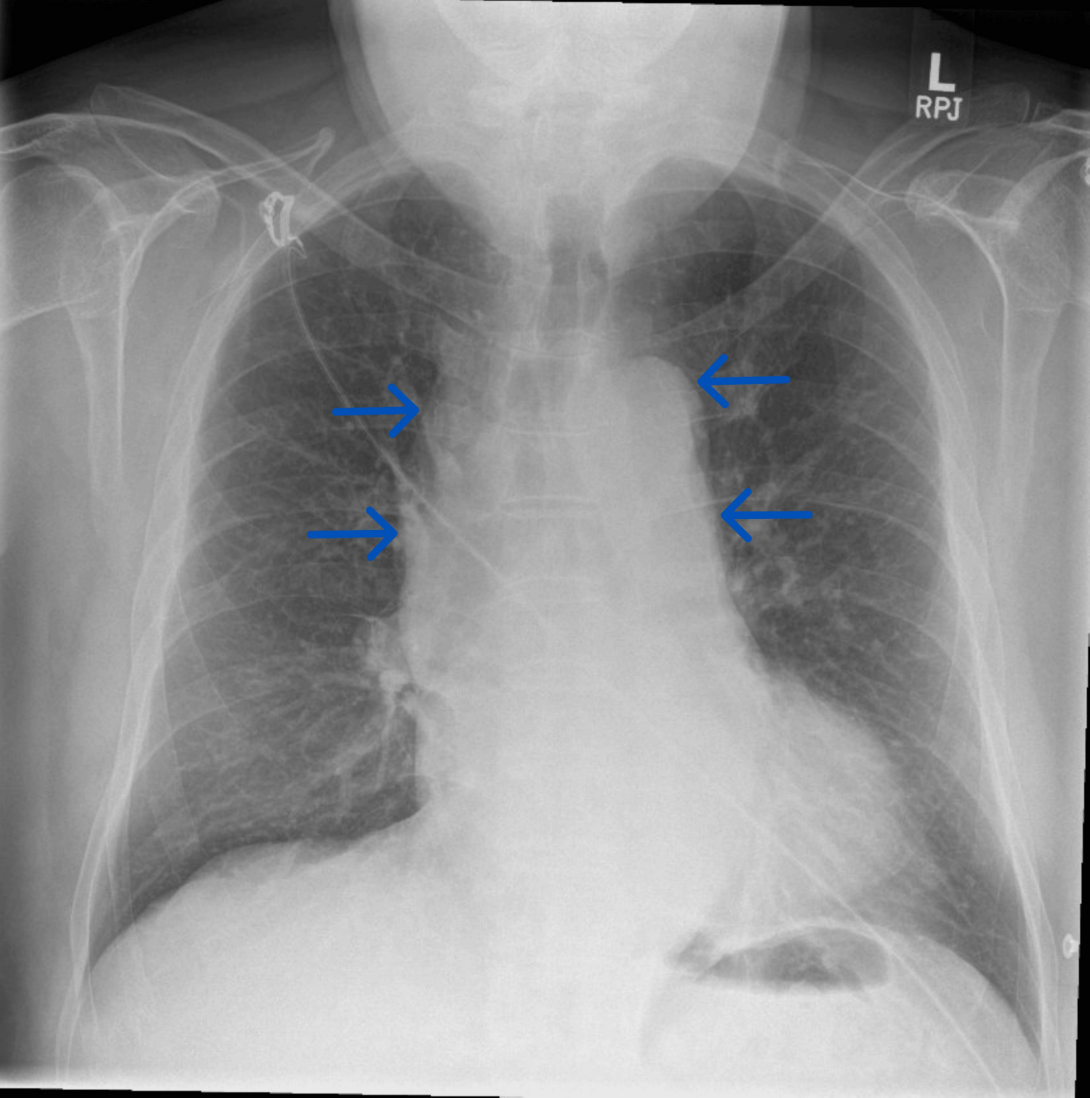
Chest radiograph at hospital admission. A chest radiograph obtained at admission showing a widened mediastinum (blue arrows), initially thought to indicate aortic unfolding from longstanding systemic hypertension.

A computed tomography (CT) scan of the head showed no acute intracranial abnormality. Electrocardiography (ECG) (Figure 1) showed sinus rhythm, large QRS voltages, and repolarisation abnormalities in the anterolateral and inferior leads. Chest radiography (Figure 2) demonstrated a widened mediastinum. His venous blood gas (VBG) analysis showed a lactate of 2.5 mmol/L (reference: 0.5-1.6 mmol/L). His blood glucose, electrolytes, and inflammatory markers were normal. Table 1 outlines his key laboratory investigations and their results.

**Table 1:** Summary of key laboratory investigations and results. hs-TnT: high-sensitivity troponin T; pCO_2_: partial pressure of carbon dioxide; pO_2_: partial pressure of oxygen; sO_2_: oxygen saturation.

Laboratory test	Result	Unit	Reference range
Blood count			
White blood cell count	10.7	× 10^9^/L	4.0-11.0
Haemoglobin	139	g/L	130-180
Platelet count	215	× 10^9^/L	150-400
Blood biochemistry			
Sodium	142	mmol/L	133-146
Potassium	3.8	mmol/L	3.5-5.3
Calcium (adjusted)	2.39	mmol/L	2.20-2.60
Phosphate	1.15	mmol/L	0.80-1.50
Magnesium	0.84	mmol/L	0.70-1.00
Urea	8.3	mmol/L	2.5-7.8
Creatinine	104	μmol/L	58-110
C-reactive protein	< 5.0	mg/L	< 5
Bilirubin	20	μmol/L	< 21
Protein	62	g/L	60-80
Albumin	39	g/L	35-50
Globulin	23	g/L	
Alkaline phosphatase	131	U/L	30-130
Alanine transaminase	23	U/L	< 50
Lactate	2.8	mmol/L	0.5-2.2
hs‑TnT (9 hours)	25	ng/L	< 14
Ethanol	< 101	mg/L	
Coagulation screen			
Prothrombin time	10.7	s	9.0-12.5
Activated partial thromboplastin time	24.2	s	22.1-30.9
Fibrinogen	3.4	g/L	2.0-4.0
Venous blood gas			
pH	7.37		
pCO_2_	6.65	kPa	
pO_2_	5.65	kPa	
Base excess	3.4	mmol/L	
Bicarbonate	28.7	mmol/L	
sO_2_	76.5	%	
Oxyhaemoglobin	74.9	%	
Carboxyhaemoglobin	1.7	%	< 2.0
Methaemoglobin	0.4	%	< 1.5
Glucose	5.4	mmol/L	4.0-11.1
Lactate	2.5	mmol/L	0.5-1.6

The working diagnosis was an acute neurological event, possibly a non-convulsive seizure with a post‑ictal state. A hyperacute stroke was considered; however, he had no focal neurological deficit, and his head CT showed no haemorrhage or early subtle features of ischaemia, such as loss of cortical grey-white matter differentiation, loss of the insular ribbon, obscuration of the lentiform nucleus, cortical sulcal effacement, subtle parenchymal hypodensity, or a hyperdense basilar, middle cerebral, or internal carotid artery. His full recovery of consciousness within an hour made a transient ischaemic attack (TIA) more likely than an established stroke. In the absence of headache, neck pain, or focal neurological deficit, there was no clinical suspicion of carotid or vertebrobasilar dissection; therefore, CT angiography of the extracranial and intracranial arteries from the aortic arch to the skull vertex was not pursued. Cardiac pathology was also considered, given the possible cardiac arrest at the onset of his symptoms. However, a cardiac diagnosis was deemed less likely because of the prolonged altered consciousness and the absence of ongoing haemodynamic instability or significant arrhythmia on arrival to hospital. There were no prior imaging studies for comparison, and the ECG and chest radiograph abnormalities (Figures 1, 2) were attributed to left ventricular hypertrophy and aortic unfolding, respectively, secondary to longstanding hypertension. In view of the working diagnosis, further assessment with head magnetic resonance imaging (MRI) and electroencephalography (EEG) was planned. However, these were not urgently available as he presented on a bank holiday.

**Figure 3:**
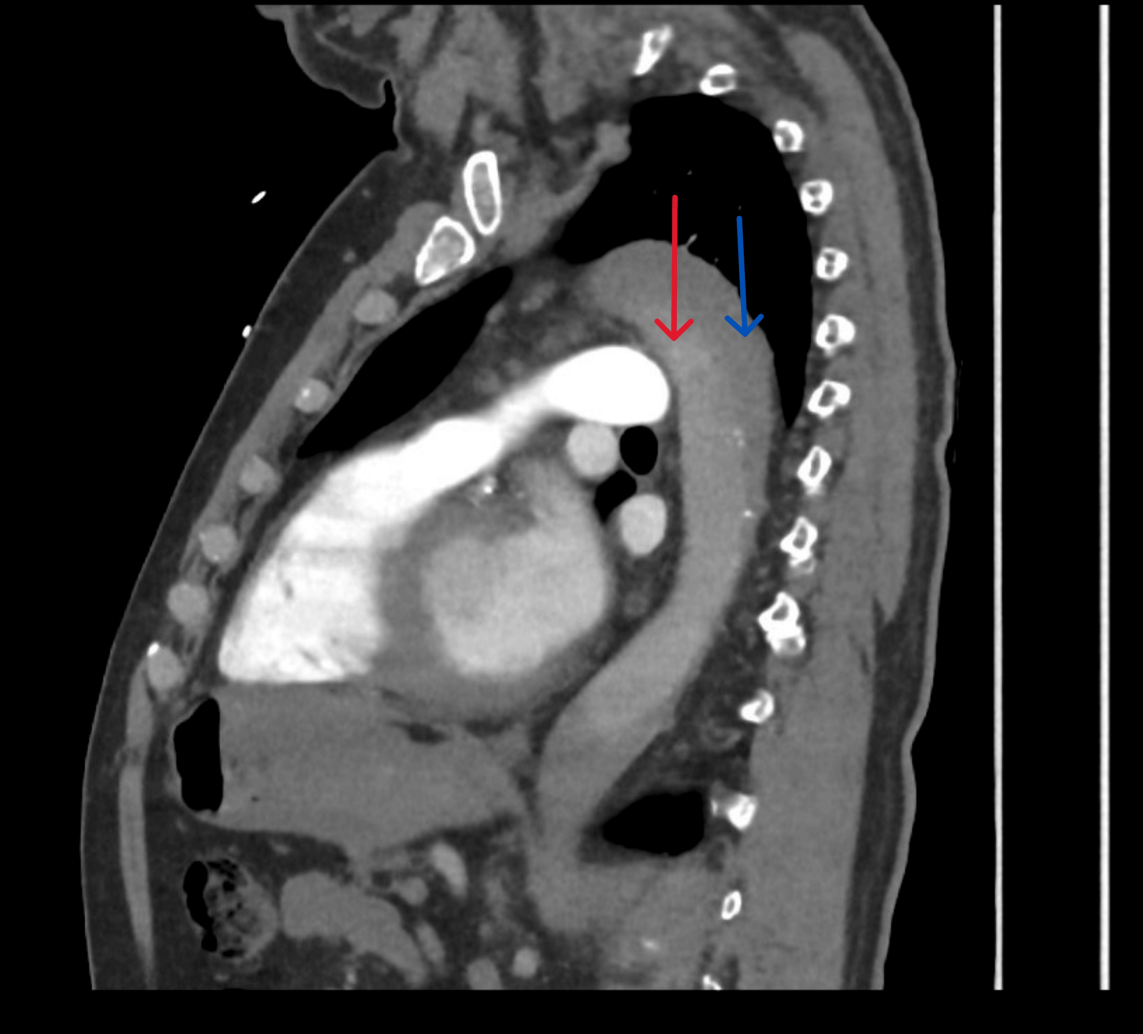
CTPA in sagittal view. Computed tomography pulmonary angiogram (CTPA) in sagittal view showing the dissected aorta. There is no contrast opacifying the aorta because this computed tomography (CT) scan was performed as a dedicated pulmonary embolism study, with contrast timing optimised for the pulmonary arterial circulation. Red arrow: true lumen; blue arrow: false lumen.

**Figure 4:**
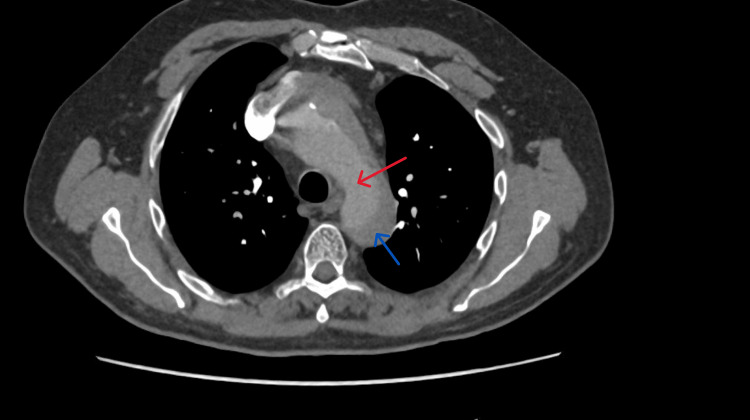
CTPA in axial view. Computed tomography pulmonary angiogram (CTPA) in axial view showing the dissected aortic arch. There is no contrast opacifying the aortic arch because this computed tomography (CT) scan was performed as a dedicated pulmonary embolism study, with contrast timing optimised for the pulmonary arterial circulation. Red arrow: true lumen; Blue arrow: false lumen.

He was admitted for observation with continuous cardiac monitoring. Overnight, his vital signs were stable, and his ECG repolarisation abnormalities also remained stable, with no dynamic evolution. High‑sensitivity troponin T, measured approximately nine hours after the onset of altered consciousness, was 25 ng/L (reference: <14 ng/L) (Table 1). Later in the morning, his pulse oximeter detected a desaturation to 80% on ambulation. Given a 6.5‑hour car journey the day before presentation, pulmonary embolism was suspected, and a computed tomography pulmonary angiography (CTPA) was requested. Instead, this revealed a TAAD extending from the aortic root to the level of the renal arteries (Figures 3, 4). Consequently, a diagnosis of acute TAAD was established approximately 14 hours after hospital arrival.

He was immediately transferred to a tertiary centre, where he underwent an emergency Bentall procedure comprising aortic root and hemiarch replacement, metallic aortic valve replacement, and reimplantation of the coronary buttons. His preoperative transoesophageal echocardiogram had confirmed severe aortic regurgitation (AR). His postoperative course was complicated by multiple bilateral embolic brain infarcts, seizures, and a prolonged period of stroke rehabilitation. He was discharged more than two months after his initial admission. His imaging studies during hospitalisation and their key findings are summarised in Table 2.

**Table 2:** Summary of imaging studies and their key findings. AR: aortic regurgitation; CT: computed tomography; CTPA: computed tomography pulmonary angiography; LA: left atrial; LCC: left coronary cusp; LMS: left main stem; LV: left ventricular; MRI: magnetic resonance imaging; MV: mitral valve; RCC: right coronary cusp; RVSP: right ventricular systolic pressure; SMA: superior mesenteric artery; TAAD: type A aortic dissection; TV: tricuspid valve.

Imaging study	Key findings
CT of the head (non‑contrast)	Normal skull.
	Mild cerebral involutional changes.
	Normal gyral and sulcal patterns.
	Preserved grey-white matter differentiation.
	No evidence of acute infarction.
	Mild chronic small vessel ischaemic change.
	No intracranial haemorrhage or space-occupying lesion.
Chest radiography	Widening of the mediastinal silhouette.
	Borderline cardiomegaly.
	Clear lung fields with no evidence of consolidation, space‑occupying lesion, pulmonary oedema, or pleural effusion.
CTPA	No evidence of pulmonary embolism or acute right‑heart strain.
	Stanford TAAD extending from the aortic root to the level of the renal arteries.
	Cardiomegaly and aneurysmal dilatation of the ascending aorta (ascending aorta diameter: 56 mm).
	The brachiocephalic trunk, coeliac trunk, and SMA are supplied by both the true and false lumens.
	The left common carotid, left subclavian, left renal, and right renal arteries are supplied by the true lumen only.
	No mediastinal haematoma, pericardial effusion, pulmonary infarction, pleural effusion, pneumothorax, or haemothorax.
	Bilateral acute anterior buckle rib fractures: right 4th and 5th ribs, and left 4th to 6th ribs.
Transoesophageal echocardiography (preoperative)	Moderate dilatation of the aorta from the root through the descending thoracic segment, with an intimal flap visualised throughout, consistent with a TAAD.
	Severe AR secondary to prolapse of the LCC.
	Moderate LV dilatation with mild to moderate impairment of systolic function.
	The LMS arises from the true lumen.
	The RCC is not visualised.
	No LA thrombus.
	Normal MV structure and function.
	Grossly normal TV and RVSP.
	Limited visualisation of right‑sided cardiac structures due to enlargement of the aortic root and ascending aorta from the dissection.
MRI of the head (postoperative day 13)	Multiple tiny bilateral cerebral and cerebellar foci of restricted diffusion.
	Widespread foci of microhaemorrhages throughout the brain parenchyma.
	Overall appearances are suggestive of multiple bilateral embolic infarcts.

## Discussion

Our patient presented with a one‑hour episode of altered consciousness. At symptom onset, he became suddenly vacant and unresponsive, and was reportedly pulseless, raising the possibility of an out‑of‑hospital cardiac arrest. His vital signs remained stable during hospital observation, but a desaturation on ambulation prompted a CTPA, which demonstrated a TAAD approximately 14 hours after symptom onset. Continuous cardiac monitoring from presentation through several postoperative days did not reveal any significant or life‑threatening arrhythmias. Findings from the history, examination, and initial investigations did not support hypertensive, metabolic, toxic, or infective encephalopathy as the cause of his altered consciousness. The TAAD identified on CTPA could be explained by one of two possible mechanisms. Trauma is a recognised risk factor for AAD [10], and the dissection may have resulted from chest compressions delivered during attempted resuscitation at home. However, this mechanism would not account for the initial vacant, unresponsive episode that prompted the resuscitation attempt. Moreover, although he sustained buckle fractures of the anterior ribs, there was no evidence of injury to other mediastinal structures or the lungs on CTPA or at surgery. Alternatively, an acute TAAD may have been the primary event, providing a coherent and unifying explanation for all his presenting features. As previously noted, AAD can be rapidly fatal and, as will be discussed subsequently, altered consciousness may itself be a manifestation of AAD. Although the precise sequence of events in our patient cannot be definitively established, the overall clinical picture more strongly supports acute TAAD as the initiating event.

Pain in the chest, back, and/or abdomen is reported in over 90% of cases of AAD [2,3,8,10]. Painless presentations are therefore uncommon and pose a significant diagnostic challenge. In our patient, the absence of pain contributed to an initial misdiagnosis in the ED, and the correct diagnosis of acute TAAD was made incidentally after a significant delay. Our patient’s demographic and clinical profile aligns with known epidemiological patterns. The mean age of AAD onset is 63 years [1], and approximately two‑thirds of patients are male [3,8]. Systemic hypertension is the most common predisposing factor, associated with AAD in approximately three-quarters of cases [3,8]. A wide range of genetic, acquired, and iatrogenic conditions also increase aortic wall vulnerability, including Marfan syndrome, Ehlers-Danlos syndrome, osteogenesis imperfecta, Turner syndrome, Noonan syndrome, Loeys-Dietz syndrome, bicuspid aortic valve, coarctation of the aorta, autosomal dominant polycystic kidney disease, aortic aneurysm, aortic atherosclerosis, pregnancy, syphilitic aortitis, connective tissue disorders, cocaine use, aortic or mitral valve surgery, coronary artery bypass grafting, and cardiac catheterisation.

Complications significantly influence the clinical presentation of AAD. Acute AR is particularly common in acute TAAD, occurring in up to three‑quarters of cases [10]. Although AR may be asymptomatic, it can precipitate heart failure, pulmonary oedema, or cardiogenic shock. Pericardial effusion occurs in approximately one‑third of acute TAAD patients due to transudation from the false lumen and is usually haemodynamically insignificant [10]. However, rupture may lead to haemopericardium and cardiac tamponade.

End‑organ malperfusion is another major complication and typically results from obstruction of branch vessels by the dissection flap [10]. However, thromboembolism, compression by the expanding false lumen, or leakage into adjacent tissues may also contribute [10]. Malperfusion can manifest as acute coronary syndrome, syncope, altered consciousness, coma, TIA, ischaemic stroke, spinal ischaemia, peripheral neuropathy, mesenteric ischaemia or infarction, renal ischaemia or infarction, acute kidney injury, or acute limb ischaemia [1,10]. Syncope may also arise from severe AR, ventricular outflow obstruction, cardiac tamponade, aortic baroreceptor activation, pain-induced vasovagal reflex, or hypovolaemia from false lumen rupture [10].

Additional thoracic complications include pleural effusions, which may be small and inflammatory or large haemorrhagic effusions due to aortic leakage; dyspnoea from pulmonary artery compression or aortopulmonary fistula formation; and haemoptysis from lung compression or rupture into the lung [10]. Compression of mediastinal structures may result in Horner syndrome, superior vena cava syndrome, dysphagia, or hoarseness. Rarely, gastrointestinal haemorrhage occurs due to mesenteric infarction, aorto‑oesophageal fistula, or rupture of the false lumen into the proximal small intestine [10].

Approximately 6.4% of AAD cases are painless, and complications often dominate the clinical picture [2]. In our patient, altered consciousness was the principal presenting feature, consistent with cerebral malperfusion. Manifestations of cerebral malperfusion, including alterations in consciousness and focal neurological deficits, are the most common presentations of painless AAD [9,11,12]. Painless AAD is also associated with older age, Marfan syndrome, steroid therapy, TAAD, and iatrogenic dissection [1,2,10]. Park et al. [2] reported that nearly three‑quarters of painless cases in the International Registry of Acute Aortic Dissection (IRAD) were type A, while Marroush et al. [11] found that 88% of painless cases in their systematic review were type A. Acute TAAD may involve the head and neck vessels or compromise the flow through them, resulting in syncope, altered consciousness, or neurological deficits - clinical features that may also impair pain perception [13]. In acute TAAD, increased pressure on the aortic arch and carotid sinuses may stimulate their mechanoreceptors, leading to baroreflex‑mediated bradycardia and/or hypotension [12]. This may explain our patient's bradycardia and relative hypotension at presentation, although he was on antihypertensive therapy that included a rate‑limiting medication.

Physical examination may reveal pulse deficits or an interarm systolic BP difference greater than 20 mmHg. Pulse deficits occur in a minority of AAD cases, seen in about 30% of TAAD and 20% of type B aortic dissection (TBAD) presentations [3,8]. Hence, the absence of pulse deficits does not exclude a diagnosis of AAD. Elevated BP at presentation is more common in acute TBAD [3,8,10]. Approximately one‑fifth of AAD patients present with hypotension or shock, often reflecting end‑organ complications or aortic complications such as rupture or true lumen compression [10]. In our patient, the absence of clinical suspicion led to the omission of assessment of pulse deficits and interarm systolic BP differences. Although his daughter’s inability to palpate a carotid pulse at home suggests a cardiac arrest, it is also possible that this represented a pulse deficit. A diastolic murmur of AR is present in only two‑fifths of acute TAAD cases at presentation [3]. Therefore, as with pulse deficits, its absence does not exclude an AAD. Diastolic murmurs can be difficult to appreciate clinically, which likely explains why no murmur was heard from our patient at presentation despite subsequent echocardiographic evidence of severe AR. His wide pulse pressure, however, was consistent with the acute AR.

D‑dimer levels are typically elevated in AAD, although they may also be increased in other conditions such as venous thromboembolism, infection, inflammation, cancer, pregnancy, recent surgery, or trauma. Despite this lack of specificity, the D‑dimer test has a very high negative predictive value in evaluating suspected AAD cases, with normal levels making the diagnosis unlikely [1]. AAD commonly causes a widened mediastinum on chest radiography, yet more than 20% of cases show no abnormality in the mediastinum or the aortic silhouette [3]. ECG abnormalities occur in approximately 30% of AAD cases, reflecting coronary malperfusion, pre‑existing systemic hypertension, or underlying coronary artery disease [3]. ECG‑gated cardiovascular CT from neck to pelvis is the recommended imaging modality for diagnosing AAD and assessing complications [1]. Our patient’s ECG demonstrated ST-T wave abnormalities, which were attributed to longstanding essential hypertension. In the absence of previous ECGs for comparison, however, and given his elevated troponin level, coronary malperfusion cannot be excluded. His painless presentation lowered clinical suspicion for AAD, and the widened mediastinum on his chest radiograph was also misattributed to hypertensive changes.

Surgical intervention is the standard of care for acute TAAD, whereas uncomplicated acute TBAD is generally managed medically [1]. Endovascular or surgical intervention is indicated in acute TBAD when complicated by malperfusion, rupture, refractory pain, or uncontrolled hypertension [1]. Regardless of subtype, pain management and optimal control of BP and HR are essential in the acute management of AAD [1].

Many case reports describe painless AAD presenting with nonspecific symptoms such as anorexia [14], nausea [14], vomiting [14], dyspepsia [14], abdominal discomfort [14], diaphoresis [14], fatigue [14,15], generalised weakness [14], and fever [15], or with features of end‑organ involvement such as syncope [14], altered mental status [12], focal neurological deficits [12,14], hoarseness [16], cough [15], haemoptysis [15,17], dyspnoea [11,15], palpitations [15], melaena [14], or haematochezia [14]. As in our case, the absence of pain in these cases frequently led to delayed or missed diagnosis. Asymptomatic, incidental dissections have also been reported, often discovered during imaging for apparently unrelated findings such as new AR [18], ECG abnormalities [19], or groin swelling [20].

## Conclusions

Painless AAD remains a rare but important diagnostic pitfall. The absence of characteristic pain can lower clinical suspicion and divert clinicians toward alternative diagnoses, increasing the risk of morbidity and mortality. Painless AAD is most often type A, and this case reinforces the need to consider AAD in any patient presenting with acute or sudden‑onset neurological impairment, whether global (such as coma, altered consciousness, or syncope) or focal, regardless of the presence or absence of pain. In such presentations, failure to confirm more common neurological or cardiac diagnoses after initial evaluation should prompt clinicians to consider AAD and pursue further assessments, including checks for pulse deficits and significant interarm (or interlimb) systolic BP differences, alongside ECG, chest radiography, and D‑dimer testing. Early diagnosis and timely management remain essential to improving outcomes. Importantly, in patients presenting with focal neurological deficits suggestive of acute ischaemic stroke, unrecognised underlying AAD carries a significant risk of harm from fibrinolytic therapy.
